# An analysis of central venous catheter-related bloodstream infections in patients treated in a Swedish Covid-19 intensive care unit

**DOI:** 10.1177/20503121241233213

**Published:** 2024-04-15

**Authors:** Petter Lunnemar, Knut Taxbro, Fredrik Hammarskjöld

**Affiliations:** 1 Department of Anaesthesiology and Intensive Care Medicine, Ryhov County Hospital, Jönköping, Sweden; 2 Faculty of Medicine and Health Sciences, Linköping University, Linköping, Sweden; 3 Department of Biomedical and Clinical Sciences, Linköping University, Linköping, Sweden

**Keywords:** Central venous catheter, catheter-related bloodstream infection, Covid-19, SARS-CoV-2, intensive care

## Abstract

**Background:**

Catheter-related bloodstream infection is a well-known, severe complication of central venous catheter insertion. Studies that have evaluated the coronavirus disease 2019 pandemic’s influence on the incidence of catheter-related bloodstream infection in intensive care units are limited. Therefore, we conducted a retrospective study on catheter-related bloodstream infection in coronavirus disease 2019 intensive care unit with previously documented low incidence rates to evaluate the pandemic’s impact.

**Objectives:**

To evaluate the impact of the coronavirus disease 2019 pandemic on catheter-related bloodstream infection incidence in the intensive care unit.

**Methods:**

All central venous catheter-inserted patients aged ⩾18 years admitted to the intensive care unit with coronavirus disease 2019 pneumonia were included. The primary outcome was the incidence of catheter-related bloodstream infection, and the secondary outcome was the detection of catheter-related bloodstream infection-causative microorganisms.

**Results:**

During the pandemic’s first year, 124 patients were admitted, and 203 central venous catheters were inserted. Two patients developed catheter-related bloodstream infection. The incidence of catheter-related bloodstream infection was 0.79/1000 catheter days. The microorganisms responsible for catheter-related bloodstream infection were *Staphylococcus epidermidis* and *Escherichia coli.*

**Conclusion:**

This study revealed a low incidence of catheter-related bloodstream infection in the coronavirus disease 2019-intensive care unit, thus suggesting that coronavirus disease 2019 is not a risk factor for catheter-related bloodstream infection and indicating the high resilience of well-established routines aimed at catheter-related bloodstream infection prevention.

## Introduction

Central venous catheter (CVC)-related bloodstream infection (CRBSI) is a well-known complication of CVC usage.^1,2^ Structured and meticulous adherence to well-established routines for CVC insertion and care can help maintain low long-term CRBSI incidence rates.^3,4^

The coronavirus disease 2019 (Covid-19) pandemic has imposed a massive burden on healthcare systems worldwide, particularly intensive care units (ICUs). Data from the *WHO-China Joint Mission on COVID-19* in February 2020 revealed that 6.1% of all Covid-19-infected patients develop critical disease that demands intensive care.^5^

Several risk factors for increased hospital-acquired infections (HAIs) in patients with Covid-19 on invasive mechanical ventilation (IMV) have been proposed, such as prolonged IMV, the prone position, steroid treatment, severe mucus production, Covid-19’s effect on the immune system, ICU understaffing and inexperienced staff.^6–11^ Several studies have reported an increased risk of ventilator-associated pneumonia (VAP).^7,12^ Understaffed ICUs and inexperienced staff potentially lead to lower compliance with basic hygiene procedures and other specific measures for the prevention of HAIs.^6,13^ Studies evaluating overall bloodstream infections, including CVC-related infections, in patients with Covid-19 have yielded conflicting results.^14–17^ Several reasons for this phenomenon exist: case-mix, adherence to prevention strategies, and definitions used, among others.

Some of the risk factors associated with HAI in the Covid-19-ICU (C-ICU) could theoretically increase the risk off CRBSI. However, CRBSI in the C-ICU has been examined to considerably lesser extent than VAP.^18^

The Ryhov County Hospital ICU, which employs an active intravascular team, has for many years managed to maintain low CRBSI incidence rates.^3,19,20^ The dedicated team, including anaesthesiologists and ICU-nurses, is responsible for all routines, implementations and evaluation of complications related to CVC insertion and use. Therefore, this study aimed to evaluate the incidence of CRBSI in the C-ICU during the first year of the pandemic.

## Methods

This was a retrospective, cohort study. Data on all C-ICU patients with Covid-19 pneumonia requiring ICU care between 14 March 2020 and 13 March 2021, were collected.

The hospital’s ICU is a seven-bed mixed ICU with approximately 500 patients admitted annually.

All employed physicians are board-certified specialists in anaesthesiology and intensive care medicine or are under training. They performed all CVC insertion prior to and during the pandemic. CVCs were always inserted with ultra-sound guidance both pre and during Covid-19-pandemic. All the nurses are registered ICU nurses. In response to the Covid-19 outbreak, the demand for ICU beds increased rapidly, resulting in the temporary opening of a C-ICU with up to 12 ventilator beds located in a postoperative unit. The required number of ICU nurses was not achieved; therefore, nurse anaesthetists were recruited to the C-ICU. The C-ICU was shifted to various locations in the hospital as patient inflow fluctuated.

### Patients

All CVC-inserted patients aged ⩾18 years admitted to the ICU with Covid-19-associated pneumonia were included. CVC types other than non-tunnelled single, multi-lumen or haemodialysis catheters were excluded.

Each patient was followed up during ICU stay. A patient who was readmitted to the ICU during their hospital stay had all their ICU admissions included.

### Central venous catheters

All CVCs were inserted by an anaesthesiologist using maximal sterile precautions (sterile gloves, masks, caps and large drapes). No prophylactic antibiotics were administered prior to CVC insertion. Before insertion, the skin was cleaned and dried using a solution containing 0.5% chlorhexidine (wt/vol) in 70% alcohol (SCHA). The following CVCs were used: a pressure-injectable quad-lumen central venous catheterization kit, Arrowg + ard Blue Plus™ (Arrow International, Inc. Reading, PA, USA), Certofix^®^ Mono V 320 (B. Braun Melsungen AG, Melsungen, Germany), and a Gambro GamCath Dolphin Protect–High-flow double-lumen catheter kit (Gambro Kathetertechnik Hechingen, Hechingen, Germany). When the catheter was inserted, it was secured with monofilament sutures and covered with a semipermeable dressing (Tegaderm HP™; 3M Healthcare, St. Paul, MN, USA). CVC use and care is performed by all ICU-nurses. However, during the pandemic, nurse anaesthetists, who normally use CVCs but do not perform the CVC care also took part in the CVC care. Every third day, the insertion site was cleaned with SCHA, and injection membranes, stopcocks, and dressings were changed. From day three after CVC insertion, a dressing containing chlorhexidine gel was used (Tegaderm CHG™, 3M Healthcare, St. Paul, MN, USA).

### Severe acute respiratory syndrome coronavirus 2 diagnosis

Covid-19 infections were diagnosed from clinical samples (nasopharyngeal swab samples or bronchoalveolar lavage) using polymerase chain reaction testing.

### CVC cultures

As per clinical practice, all CVC tips were systematically cultured upon removal. The insertion site was cleaned with SCHA and dried before CVC removal. The distal 5 cm of the CVC was cut off, placed in a sterile tube, and sent to the local laboratory for processing using a semi-quantitative roll-plate technique.^21^ The plate was subsequently incubated at 36°C in a CO_2_ incubator (BACT/ALERT^®^; bioMériuex, Inc., Durham, NC, USA). Cultures without microbiological growth within 48 h were considered negative. Positive tip cultures were defined as ⩾15 colony-forming units (CFUs). The microorganism species were identified by mass spectrometry using matrix-assisted laser desorption/ionisation–time of flight spectrometry (MALDI-TOF) (biotype Sirius and Bioflex; Bruker Corp, Billerica, MA, USA). All cultures were analysed to map antibiotic sensitivity according to Swedish standards.^22^

On suspicion of CRBSI, blood cultures were obtained from the peripheral veins and all CVC lumens. The bottles were incubated in the BACT/ALERT 3D instrument for up to 6 days.

### Primary and secondary outcomes

The primary outcome was the incidence of CRBSI, and the secondary outcome was the detection of CRBSI-causative microorganisms.

### Data collection

Patients were identified using the ICU patient data management system (Metavision™, version 5.46, iMDsoft, Israel). Those who met the inclusion criteria had their data extracted from the hospital’s electronic file system (Cosmic™, version R8.2.07, Cambio Healthcare Systems AB, Sweden). Baseline demographics, treatment, and patient characteristics during ICU stay (except for systemic inflammatory response syndrome (SIRS)) were collected by the Jönköping County Covid-19 Research Group. CVC-specific, SIRS, and microbiological data were obtained by the first author (PL). Any uncertainties regarding the collected data were reviewed and discussed by the Covid-19 Research Group.

Patient baseline characteristics included age, sex, smoking status, body mass index, immunosuppression, and solid or haematologic malignancies (active or past). Comorbidities were defined using the International Classification of Diseases and Related Health Problems, tenth revision, (ICD-10) codes in patients’ medical journals.^23^ Index data codes were as follows: cardiovascular disease (I25 and/or I50); hypertension (I10); diabetes (E10 and/or E11); asthma (J45); chronic obstructive pulmonary disease (J44); pulmonary disease (J43) and/or pulmonary fibrosis (J84); chronic liver disease (K70–K77); and neuromuscular disease, defined as post-polio syndrome or multiple sclerosis (B91, G14 and/or G53). Other data associated with ICU stay included the acute respiratory distress syndrome (ARDS) score; ARDS reason; simplified acute physiology score 3 (SAPS 3); SIRS; haemofiltration/dialysis; high flow nasal oxygen; inotropes/vasoactive agents; intubation; total stay in the ICU, and mortality counted as days from admittance to the ICU. The following CVC data were included: hospital where the CVC was inserted and removed, date of insertion and removal, vein and CVC type. The following microbiological data were analysed: blood cultures from both CVC lumens, peripheral blood cultures and tip cultures.

All patients with positive blood and/or catheter tip cultures were diagnosed with CRBSI by PL and FH in coherence.

### Definitions

CRBSI was defined as a positive peripheral blood culture with a positive CVC blood culture or catheter tip culture with indistinguishable microorganisms (same species and antibiogram) and SIRS without any other source of infection. CVC colonisation was defined as a positive culture from the catheter tip with at least 15 CFUs and no symptoms of CRBSI.^21^

Multidrug-resistant microorganisms were defined as meticillin-resistant *Staphylococcus aureus*, vancomycin-resistant enterococci, extended-spectrum beta-lactamase (ESBL), and extended spectrum beta-lactamase with carbapenemase activity (ESBL-carba).

The definitions of ARDS,^24^ SAPS 3,^25^ SIRS^26^ and immunosuppression^25^ are shown in the Appendix 1.

### Ethical considerations

This study was approved by the Swedish Ethical Review Authority on 26 August 2020 (DNR: 2020-02758). The study’s design, being retrospective in nature, rendered patient consent unnecessary since patient treatment would not be influenced. The requirement for written informed consent was waived by the Swedish Ethical Review Authority.

### Statistical analysis

All data were compiled in a database (Excel, version 2016).

Statistical analyses were performed using IBM SPSS Statistics software (version 28.0; Armonk, NY, USA). Descriptive statistics were used, with categorical variables presented as frequencies (percentages) and continuous variables as medians (interquartile ranges (IQRs)).

## Results

### Patients

A total of 134 patients were admitted to the C-ICU with Covid-19 pneumonia, and 124 met the inclusion criteria. Seventeen of the 124 patients were admitted more than once, resulting in a total of 141 ICU admissions. IMV was used in 111 (89.5%) ICU stays, and 111 (89.5%) were classified as ARDS (Table 1). The overall 90-day mortality rate was 14 (11.3%).

**Table 1.** Patient and treatment characteristics..

	Total
Patients	124
Age (years), median **(**IQR**)**	64 (54.0–72.8)
Sex, *n* (%)	
Male	88 (71.0)
Female	36 (29.0)
BMI, median **(**IQR**)**	29 (27.0–33.0)
Missing	2
Cardiovascular disease, *n* (%)	27 (21.8)
Hypertension *n* (%)	66 (53.2)
Diabetes, *n* (%)	
Type 1	4 (3.2)
Type 2	30 (24.2)
Liver cirrhosis, *n* (%)	2 (1.6)
Neuromuscular disease, *n* (%)	8 (6.5)
Immunosuppression, *n* (%)	17 (13.7)
Asthma, *n* (%)	18 (14.5)
COPD, *n* (%)	15 (12.1)
Other lung disease, *n* (%)	16 (12.9)
Smoking status, *n* (%)	41 (33.1)
Cancer, *n* (%)	15 (12.1)
Haematologic cancer, *n* (%)	3 (2.4)
Total ICU admissions, *n*	141
Total ICU stay, *n* (days)	2120
Total ICU stay per admission, median (days) **(**IQR**)**	11 (4.0–19.5)
Readmissions, *n*	17
SAPS 3 score, median **(**IQR**)**	57 (49.0–63.8)
Intubated, *n* (%)	111 (89.5)
HFNO, *n* (%)	83 (66.9)
Noradrenaline, *n* (%)	
<0.1 µg/kg/min	81 (65.3)
>0.1 µg/kg/min	23 (18.5)
ARDS reason, *n* (%)	
Covid-19	111 (89.5)
ARDS score, *n* (%)	
Mild (P/F ratio > 26.6 kPa)	9 (7.3)
Moderate (P/F ratio > 13.3 and ⩽26.6 kPa)	87 (70.2)
Severe (P/F ratio ⩽ 13.3 kPa)	15 (12.1)
Haemofiltration/Dialysis, *n* (%)	24 (19.4)
SIRS, *n* (%)	123 (99.2)

Categorical variables are presented as frequencies (%) and continuous variables as medians (IQRs).

*n*: number; BMI: body mass index; COPD: chronic obstructive pulmonary disease; ICU: intensive care unit; SAPS 3: simplified acute physiology score 3; HFNO: high flow nasal oxygen; ARDS: acute respiratory distress syndrome; SIRS: systemic inflammatory response syndrome.

The most common comorbidities were hypertension (53.2%), type 2 diabetes (24.2%), and cardiovascular disease (21.8%) (Table 1).

### Central venous catheter

**Figure 1.**
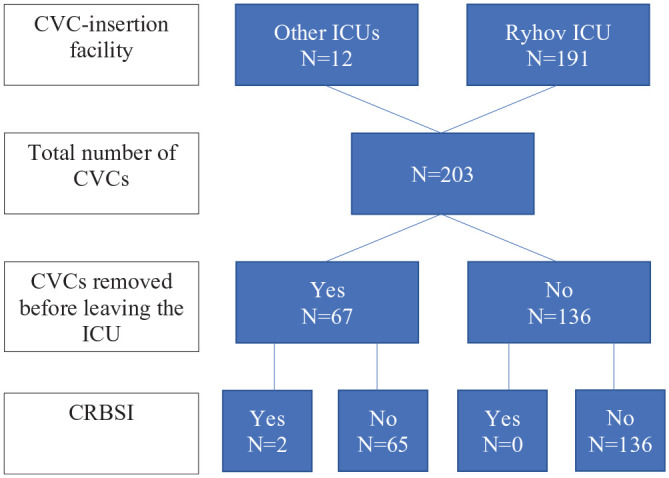
Flowchart of CVC insertion and removal. *n*: number; CVC: central venous catheter; ICU: intensive care unit; CRBSI: catheter-related bloodstream infection.

A total of 203 CVCs were inserted in the 124 patients (Figure 1). The median number of catheters per admission was one (IQR: 1.0–1.5), with a median ICU stay of 11 (IQR: 3.0–20.0) days, resulting in a total of 2542 catheter days. The most used vein was the internal jugular vein (69.5%), followed by the femoral vein (18.2%); 160 (78.8%) of the catheters were multi-lumen catheters (Table 2). Sixty-seven (33.0%) CVCs were removed during ICU stay, and all of these were sent for culture.

**Table 2.** CVC data and patient outcomes.

	Total
CVC, *n*	203
CVCs inserted per admission, *n* (IQR)	1 (1.0–1.5)
Total catheter days	2542
Median catheter days (IQR)	11 [3.0–20.0]
CVCs inserted before ICU admission, *n* (%)	12 (5.9)
CVCs removed before leaving ICU, *n* (%)	67 (33.0)
Vein, *n* (%)	
Subclavian	6 (3.0)
Femoral	37 (18.2)
Internal jugular	141 (69.5)
External jugular	4 (2.0)
Axillary	5 (2.5)
Unknown	10 (4.9)
CVC type, *n* (%)	
Non-tunnelled single-lumen catheter	5 (2.5)
Non-tunnelled multi-lumen catheter	160 (78.8)
Non-tunnelled haemodialysis catheter	38 (18.7)
CRBSI cases, *n* (%)	2 (1.6)
CRBSI per 1000 catheter-days	0.79/1000
90-days mortality, *n* (%)	14 (11.3)
90-days mortality, *n* (IQR)	29 (11.5–64.8)

Categorical variables are presented as frequencies (%) and continuous variables as medians (IQRs).

*n*: number; CVC: central venous catheter; CRBSI: catheter-related bloodstream infection.

### Catheter-related bloodstream infection

Seventeen blood cultures exhibited microbial growth. Three (4.5%) out of 67 tip cultures demonstrated significant microbial growth. Two (1.6%) patients were diagnosed with CRBSI, with a corresponding incidence of 0.79/1000 catheter days and time from CVC insertion to CRBSI diagnosis of 32 and 96 days.

The microorganisms that caused the two cases of CRBSI were *Staphylococcus epidermidis* (*S. epidermidis*) and *Escherichia coli* (*E. coli*). The two infected patients had positive blood cultures from blood drawn from the CVC and peripheral vein. CRBSI-causative *E. coli* was classified as ESBL, and the infection might have contributed to the patient’s death.

Positive microbial growth without positive blood cultures was detected in 3 of the 67 catheter tip cultures. Hence, 3 (4.5%) cases of colonisation were observed, corresponding to 1.18/1000 catheter days. The microorganisms detected in the tip cultures were all *S. epidermidis* strains.

## Discussion

This study’s main finding was a low CRBSI incidence of 0.79/1000 catheter days in C-ICU patients during the first year of the Covid-19 pandemic.

In several studies conducted over different periods of time using various vascular devices, Ryhov County Hospital has been able to demonstrate an overall low CRBSI incidence.^3,19,20,27–29^

An ICU incidence <2/1000 catheter days is reportedly difficult to achieve over long periods of time.^4^ We challenge this statement since two previous ICU studies in Jönköping have revealed CRBSI incidence rates of 1.9 and 1.4/1000 catheter days.^3,19^ The median number of catheter days was 4.0 and 8.7 days, respectively. In the present study, median days with catheter were higher, but with a narrower interval of 11 days. Despite the longer use of the CVCs, the incidence in this study is 0.79/1000 days, which indicates high adherence to hygiene routines for insertion and care of CVCs.

This study yielded a low incidence (0.79/1000 catheter days), despite the severe stress experienced by ICU staff throughout the pandemic. This indicates a high degree of resilience in the prevention of CRBSI. Several possible reasons justify this phenomenon: the continuous and diligent implementation of routine CVC insertion, removal, and care measures as well as an active CVC support team. Nurse anaesthetists do not normally care for CVCs at our hospital. Since they participated in the C-ICU work, including CVC-care, it seems that they have adopted the hygiene routines well. This indicate high adherence throughout the hospital which is also supported by a recently published study from our hospital that demonstrated a low incidence of CRBSI in oncologic patients with a subcutaneous port inserted during the pandemic.^27^

The low CRBSI incidence rates from repeated studies could have resulted from several factors related to CVC insertion and care. Structured hygiene routines and repeated training in CVC insertion and care have been implemented. CVCs are constantly evaluated with regard to their risk-benefit ratio, and they should be removed if usage is considered unnecessary. These findings are corroborated by several studies conducted in different countries and settings.^4,30–32^

The Covid-19 pandemic potentially increases HAI risk for several reasons, including a lack of sufficient infection prevention equipment, the prone position, crowded and understaffed wards, untrained staff, severe mucus production, Covid-19’s effect on the immune system, and steroid treatment.^6,7,9–11,13,33^ This is highly probable in terms of VAP.^7,12^ The abovementioned risk factors could also theoretically increase the incidence of CRBSI, that is, a lack of sufficient infection prevention equipment, Covid-19’s effect on the immune system, crowded and understaffed wards, untrained staff, and steroid treatment. However, studies that have evaluated CRBSI in the C-ICU setting are limited, and the current study could not confirm an increased incidence.

A German multicentre study evaluating ICUs found no significant increase in central line-associated bloodstream infections (CLABSIs) in ICU patients in 2019 (0.7/1000 catheter days) and 2020 (0.64/1000 catheter days).^16^ Contrarily, another study investigating CLABSI incidence revealed significant higher rates among C-ICU patients (6.31/1000 catheter days) than non-C-ICU patients (0.6/1000 catheter days).^34^ A study evaluating CLABSIs at the hospital level reported an increased incidence. CLABSI rates increased in one hospital from 1.03/1000 to 5.38/1000 catheter days and from 0.89/1000 catheter days to 3.79/1000 catheter days in another hospital. The foregoing study suggested that the reasons for these increases were the decreased use of low-risk central lines and reduced numbers of citizens with a low HAI risk seeking hospital care.^35^ Another study investigating CRBSI reported an increased incidence in patients with Covid-19 during the pandemic.^14^ The study, which was conducted at a hospital in Madrid between 2019 and 2020, showed increased CRBSI rates from 1.89/1000 to 5.53/1000 admissions.^14^ One study suggested that infection surveillance has shifted from focusing generally on infection prevention to limiting Covid-19 transmission.^36^ Furthermore, one study revealed an increased risk of overall blood stream infection rates in Covid-19-infected patients admitted to ICUs compared with that in other ICU patients; however, no significant increase in CRBSI was observed.^18^ Due to great variation in study protocols, endpoints and definitions, it is difficult to compare these studies and there is a great variation in incidences. Studies evaluating CRBSI o CLABSI incidence in COVID-19 patient with accepted endpoints are shown in Table 3.^14,16,18,34,37–41^ Our incidence is in the low range and indicates that our unit has been successful in taking care of CVCs in the C-ICU.

**Table 3.** Summary of studies evaluating central venous catheter infections in Covid-19 patients.

Author	Country	Study design	Number of CVCs, patients or device days	Endpoint	Hospital incidence	ICU incidence
Bardi et al.^38^	Spain	R cohort	140 patients*	CRBSI		17.1%^c^
Buetti et al.^8^	France	R case-control	235 patients*	CRBSI		3.4%^c^
Pérez-Granda et al.^14^	Spain	R cohort	617 CVCs**	CRBSI		6.2^b^
Soriano et al.^40^	Spain	R cohort	83 patients*	CRBSI		8.4%^d^
Verberk et al.^41^	Netherlands	R multicentre registry	367 patients*	CRBSI	11.1^a^	7.8^a^
Ben-Aderet et al.^34^	USA	R cohort	7 763 device days*** *	CLABSI		6.3^a^
Fakih et al.^37^	USA	R multicentre registry	257 898 device days***	CLABSI	0.57^a^	1.16^a^
Hlinkova et al.^39^	Slovakia	R cohort	207 patients*	CLABSI		10.6^a^
Geffers et al.^16^	Germany	R multicentre registry	696 085 patients**	CLABSI		0.64^a^

R: retrospective; CVC: central venous catheter; CRBSI: catheter-related bloodstream infection; CLABSI: central line associated bloodstream infection; USA: United States of America.

a Incidence per 1000 catheter days.

b Incidence per 1000 admission.

c Percent of cases in study group.

d Percent catheter-related bloodstream infection per 100 patients.

* Only patients with Covid-19 infection.

** Patients with or without Covid-19 infection.

*** Total days with central lines inserted with or without Covid-19 infection.

*** *Total days with central lines inserted in patients with Covid-19 infection.

Overall, these studies yielded conflicting results regarding CLABSI and CRBSI rates during the Covid-19 pandemic, in contrast to VAP where the increase was more pronounced. This indicates that patients with Covid-19 do not have an obviously greater risk of CRBSI and that maintaining a low incidence is possible, despite the challenges healthcare systems have been subjected to during the pandemic.

Owing to the limited number of cases of CRBSI and tip colonisation, drawing conclusions regarding the causative microorganisms is difficult. Our previous studies have detected similar microorganisms to those in other studies but with a considerably low incidence of multidrug-resistant bacteria. This study’s results are consistent with our previous findings.^3,19^

### Strengths and limitations

This study has several strengths. First, we conducted the study within a single unit, thus minimising the risk of differences in routines. Second, all data were collected by trained staff from the Covid-19 research group. Third, well-established routines for diagnosing CRBSI have been implemented in the department for many years. Fourth, an infectious disease specialist visited the ICU daily to evaluate and discuss infection concerns for each patient, limiting the risk of missed CRBSI diagnosis.

Notwithstanding, this study also has several limitations. First, due to this study’s retrospective, single-centre nature and involvement of a previous control group, bias and reduced validity might have existed. However, we have repeatedly reported a low incidence of CRBSI both within and outside the ICU for different vascular access systems. Second, a relatively small number of CRBSI cases was available, thus rendering a meaningful comparison between patients with and without CRBSI difficult in terms of comparison with studies from our own unit and risk factor analysis. Third, patients were not evaluated for CRBSI after leaving the ICU. Hence, an imminent CRBSI might have been overlooked. Lastly, no sample size calculation was performed since this was a cohort study evaluating all patients in the C-ICU during pandemic wave one and two.

## Conclusions

This study has revealed a low incidence of CRBSI in the C-ICU, thus suggesting that Covid-19 is not a risk factor for CRBSI and indicating the high resilience of well-established routines aimed at CRBSI prevention. Nevertheless, owing to the retrospective nature of the study and the limited number of patients included, it is not possible to definitively establish Covid-19 as a risk factor for CRBSI.

## Appendix

**Appendix 1:**

Acute respiratory distress syndrome (ARDS)^24^	Mild ARDS: 200 mmHg <Pao2/Fio2⩽ 300 mmHg or P/F ratio > 26.6 kPa (with PEEP or CPAP ⩾ 5 cmH2O, or nonventilated), or non-ventilated)
	Moderate ARDS: 100 mmHg <Pao2/Fio2⩽ 200 mmHg or P/F ratio > 13.3 and ⩽26.6 kPa (with PEEP or CPAP ⩾5 cm H2O, or non-ventilated)
	Severe ARDS: ⩽Pao2/Fio2 100 mmHg or P/F ratio ⩽ 13.3 kPa (with PEEP or CPAP ⩾5 cmH2O, or non-ventilated)
Simplified acute physiologic score 3 (SAPS 3)^25^	A scoring system with the following 20 variables: patient age, temperature, systolic blood pressure, heart rate, Glasgow coma scale, white blood cell count (g/L), platelet count (g/L), hydrogen ion concentration (pH), creatinine, bilirubin level, $PaO_{2}/FiO_{2}$ , length of stay before ICU admission, comorbidities, intrahospital location before ICU admission, use of other major therapeutic options before ICU admission, ICU admission, reason for admission, surgical status, anatomical site of surgery, and acute infection. It ranges from a theoretical minimum of zero to a maximum of 217 points
Immunosuppression^25^	A disease capable of suppressing the body’s resistance to infection, e.g., severe malnutrition, congenital immunohumoral or cellular immune deficiency; it excludes acquired immunodeficiency syndrome (AIDS) and human immunodeficiency virus (HIV) infection, metastatic cancer, haematological cancer, chemotherapy, radiotherapy, and treatment with steroids
Systemic inflammatory response syndrome (SIRS)^26^	Two or more of: Temperature > 38°C or <36°C, Heartrate > 90/min, Respiratory rate > 20/min or PaCO_2_ < 32 mm Hg (4.3 kPa), White blood cell count > 12,000/mm^3^ or <4000/mm^3^ or >10% immature bands
